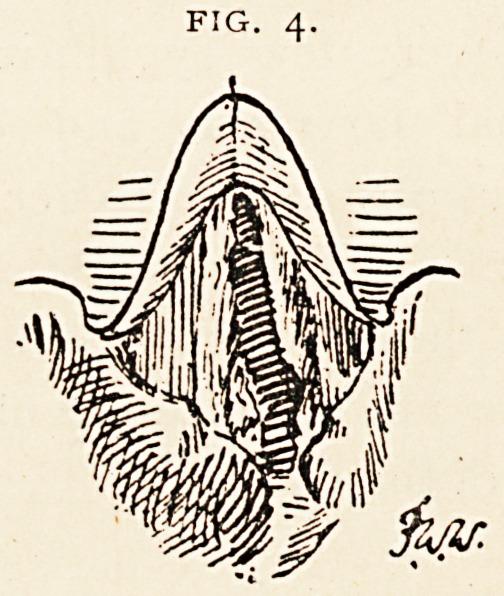# Two Cases of Intubation in Adults

**Published:** 1897-09

**Authors:** Harold F. Mole

**Affiliations:** House Physician, Bristol Royal Infirmary


### TWO CASES OF INTUBATION IN ADULTS.

BY

HAROLD F. MOLE, F.R.C.S. Eng.,
*House Physician, Bristol Royal Infirmary.*

THE necessity for intubation of the larynx in adults arises sufficiently seldom to make it worth while recording the two following cases occurring within a short time of each other.

CASE I.—M. S., aged 40, female, was admitted to the Bristol Royal Infirmary on February 12th, 1897, under the care of Dr. Shingleton Smith. She was suffering from considerable dyspnœa and pain in the larynx with troublesome cough, and her voice was reduced to a hoarse whisper. Three weeks previously she had caught cold, her voice not becoming affected, however, until a week later; but for the past week the difficulty in breathing had gradually increased. There was slight pyrexia ranging from 99° F. to 101° F. She was treated, on the assumption that the laryngeal affection was of an inflammatory nature, by leeches to the throat, diaphoretics and steam tent.

Notwithstanding this the patient's symptoms got steadily worse, paroxysmal attacks of cough with urgent dyspnœa accompanied by inspiratory stridor occurring frequently; and on the second day after admission the urgent distress of the patient necessitated immediate relief to the breathing. It was decided to attempt intubation, and this was performed with some difficulty after several unsuccessful attempts, considerable force having to be used to get the tube (the largest of O'Dwyer's set of six) through the stenosed glottis. The throat had been previously well cocainised. Considerable cough was at first caused by the presence of the tube, but later, when the patient got accustomed to it, great relief was obtained. The tube was worn for two days, during which time the patient had to be fed by nutrient enemata, as any attempt at swallowing food, either by gulping or by suction through a tube, caused a violent attack of coughing. After removal of the tube the breathing remained fairly easy, but the voice was still very husky.

An examination of the larynx on February 21st showed the following [illegible]ndition (Fig. 1): On the left a subglottic swelling is seen projecting [illegible]low the left vocal cord, which is fixed by inflammatory infiltration at the base of the left arytenoid cartilage. An extensive inflammatory mass is also seen external to the thyroid cartilage and in the pyriform fossa. The patient was given iodide of potassium in increasing doses, although there was no history of syphilis, and she continued slowly to improve, the voice, however, remaining husky.

FIG. 1.

FIG. 2.

On March 6th the laryngoscopic appearances (Fig. 2) were as follows: There is now no subglottic swelling; the left cord is fixed in the cadaveric position; and the considerable swelling below and external to the left aryteno-epiglottic fold is still very obvious.

The larynx was much the same on the patient's discharge on March 16th, and as she left the city her case could not be further traced.

This was a case of cricoid perichondritis, as evidenced by the swelling outside the aryteno-epiglottic fold and projecting below the glottis; there was also pseudo-ankylosis of the left arytenoid cartilage at the time the intubation was performed. The swelling in and around the larynx must have been much greater than is depicted in Fig. 1, as the drawing was not made until a week after the tube had been left out, the patient's breathing being then fairly easy.

The condition was probably due to cold, of which there is definite history, no evidence of syphilis being obtainable; and it appeared to be getting well without suppuration or necrosis of cartilage, which is such a common termination of these cases.

Case II.—J. N., aged 45, male, was admitted in the early morning of March 15th, 1897, under Dr. Shingleton Smith. He was suffering from extreme inspiratory dyspnœa, with sucking-in of supra-sternal and supra-clavicular regions. He was livid and covered with a clammy sweat, and his face was expressive of the greatest anxiety. There was considerable stridor, but no laryngeal excursion. He was only able to utter a few words at a time, and those in a hoarse whisper. Two days previously he had noticed "a cold in his throat," which got worse as the day went on, and during the night his breathing became difficult. Next day he consulted a doctor, who gave him a gargle, but his breathing continued to get worse until he came to the Infirmary.

As the diagnosis of the case was uncertain, a little time was expended in trying the effects of sedative draughts—cocaine spray and inhalation of nitrite of amyl: these, however, failed to give any but the slightest relief; and as he was gasping for breath it was decided to try intubation, and, failing this, laryngotomy or tracheotomy. The largest of O'Dwyer's tubes was inserted easily, with immediate and complete relief to the distressing symptoms. Ten minutes later, however, the patient coughed up the tube, notwithstanding that it appeared to have been well placed in position, and the symptoms returned at once. This occurred a second time; but on a third time inserting the tube the throat was well sprayed with cocaine to lessen the cough which immediately followed, and the patient retained the tube until the following morning (eight hours), when he again coughed it up. Although considerable dyspnœa remained, it was not enough to necessitate the use of the tube again.

The condition of the larynx after expulsion of the tube (Fig. 3) was as follows: The cords are very red and only abduct to a very slight extent, the figure showing the extreme degree of abduction. There is some subglottic swelling.

After examination of the larynx, the patient was put in a steam tent, two leeches applied to the front of the neck over the larynx and ammonium acetate given. His breathing remained very bad for a week; but five days after that he was sufficiently well to be made an

FIG. 3.

FIG. 4.

out-patient, and the condition of his larynx then was as in Fig. 4, which was taken during deep inspiration. The left cord is more movable than before. The right cord is fixed by pseudo-ankylosis of arytenoid cartilage. The right vocal process showed pachydermia with corresponding depression in the opposite cord.

An examination of the larynx on April 24th showed that it had almost completely recovered; the right cord, however, failed to reach full abduction, and there was still a little pachydermia remaining.

This case was probably an acute septic laryngitis; and the glottic obstruction at the time the intubation was performed must have been extreme, as after the tube was left out—the breathing being then much better—but a small chink remained, as shown in Fig. 3. The pachydermia may be accounted for by the fact that the man was a heavy smoker, and his occupation—a cattle drover—necessitated an excessive use of the voice.

Intubation in adults may be a simple or difficult operation, as evidenced by these two cases. Apart from the condition existing in the larynx itself, the ordinary difficulty is due to the distance from the teeth to the glottis rendering it awkward for the guiding finger to conduct the tube to its right position. This is materially aided if, at the moment the tube has cleared the epiglottis, the assistant holding the gag be requested to depress the head forwards a little, when the tube will then pass down into the larynx. With regard to feeding in these cases: some nervous patients may require nutrient enemata all the time, as occurred in the first of these; but most patients will be able to feed themselves by sucking up fluids through a glass tube, the head being placed low or on one side, as happened in the second case, or by taking some fluid into the mouth and gulping it down over the larynx.

There cannot be many conditions in which intubation in the adult will be indicated. It is only of use in temporary conditions to tide over a crisis. It will not find its application in such conditions as tubercular or cancerous disease of the larynx, and in but very few syphilitic or paralytic conditions; whereas catarrhal laryngitis and diphtheria but rarely give rise to sufficient obstruction to necessitate interference.

I am indebted to Dr. Shingleton Smith for permission to publish these two cases, and to Dr. Watson Williams for kindly allowing me to make use of sketches made by him during the course of the cases.

## Figures and Tables

**Fig. 1. f1:**
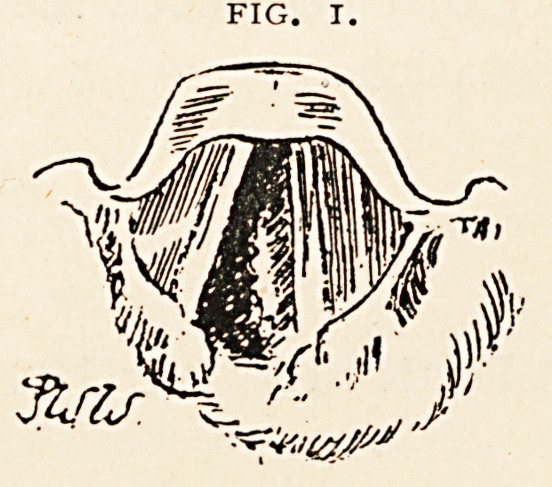


**Fig. 2. f2:**
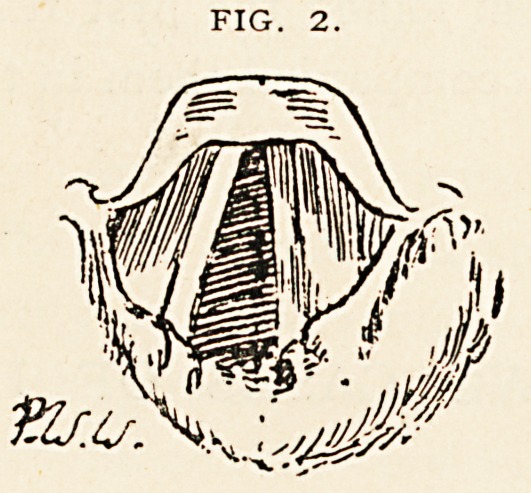


**Fig. 3. f3:**
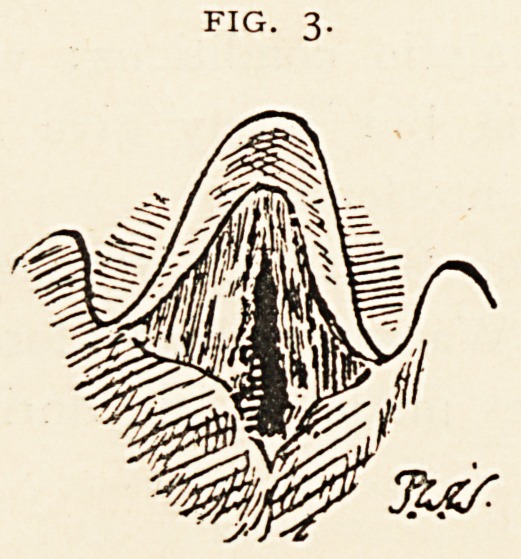


**Fig. 4. f4:**